# TLR7 Influences Germinal Center Selection in Murine SLE

**DOI:** 10.1371/journal.pone.0119925

**Published:** 2015-03-20

**Authors:** Alexis Boneparth, Weiqing Huang, Ramalingam Bethunaickan, Megan Woods, Ranjit Sahu, Shitij Arora, Meredith Akerman, Martin Lesser, Anne Davidson

**Affiliations:** 1 Center for Autoimmunity and Musculoskeletal Diseases, Feinstein Institute for Medical Research, Manhasset, New York, 11030, United States of America; 2 Biostatistics Unit, Feinstein Institute for Medical Research, Manhasset, New York, 11030, United States of America; Instituto Nacional de Ciencias Medicas y Nutricion Salvador Zubiran, MEXICO

## Abstract

TLR7 enhances germinal center maturation and migration of B cells to the dark zone where proliferation and somatic hypermutation occur. Our goal was to determine how *Tlr7* dose influences selection of the autoreactive B cell repertoire in NZW/BXSB. Yaa mice bearing the site-directed heavy chain transgene 3H9 that encodes for the TLR7 regulated anti-CL response. To create a physiologic setting in which autoreactive B cells compete for survival with non-autoreactive B cells, we generated bone marrow chimeras in which disease onset occurred with similar kinetics and the transferred 3H9+ female non-*Yaa*, male *Yaa* or male TLR7^-/Yaa^ cells could be easily identified by positivity for GFP. Deletion of 3H9 B cells occurred in the bone marrow and the remaining 3H9 follicular B cells manifested a decrease in surface IgM. Although there were differences in the naïve repertoire between the chimeras it was not possible to distinguish a clear pattern of selection against lupus related autoreactivity in TLR7^-/Yaa^ or female chimeras. By contrast, preferential expansion of 3H9+ B cells occurred in the germinal centers of male *Yaa* chimeras. In addition, although all chimeras preferentially selected 3H9/Vκ5 encoded B cells into the germinal center and plasma cell compartments, 3H9 male *Yaa* chimeras had a more diverse repertoire and positively selected the 3H9/Vκ5-48/Jκ4 pair that confers high affinity anti-cardiolipin activity. We were unable to demonstrate a consistent effect of *Tlr7* dose or *Yaa* on somatic mutations. Our data show that TLR7 excess influences the selection, expansion and diversification of B cells in the germinal center, independent of other genes in the *Yaa* locus.

## Introduction

Systemic lupus erythematosus (SLE) is an autoimmune disorder in which pathogenic autoantibodies directed to ubiquitous nuclear material initiate systemic inflammation. SLE patients have defective negative selection of autoreactive B cells at the immature and transitional checkpoints [1] and also fail to restrain pathogenic effector B cells arising in the germinal center (GC) [2–3]. Understanding how these defects contribute to pathogenic autoantibody production will allow therapy for SLE to be directed to the appropriate B cell developmental stage.

TLR7 is an endosomal TLR that recognizes single-stranded viral RNA and its expression in B cells is required for the generation of anti-RNA antibodies in SLE [4–5]. Haplodeficiency of TLR7 in SLE-prone mice bearing the Yaa locus also moderately decreases anti-DNA antibodies in addition to its effect on the anti-RNA response [6–7]. Engagement of TLR7 induces signaling through its adaptor MyD88 resulting in activation of the NFκB and Type 1 interferon pathways [8–9]. B cell intrinsic TLR signaling is amplified in GC B cells compared to follicular B cells, suggesting that TLRs play a role in the development of the antigen activated antibody repertoire [10–11]. TLR signaling drives B cells into the dark zone of the germinal center where they undergo clonal expansion, and differentiation to memory cells [12]. In accord with this data, mice with a B cell specific *Tlr7* deficiency have impaired anti-viral responses due to decreased entry of B cells into the GC dark zones where clonal proliferation and somatic mutation occur [13]. Two recent studies have shown that in lupus models the complete absence of TLR7 compromises B cell survival and abrogates spontaneous germinal center formation and the production of anti-Sm/RNP, anti-dsDNA, anti-cardiolipin (CL) and anti-nucleosome antibodies in a B cell intrinsic manner. By contrast, deficiency of MyD88 in macrophages and dendritic cells has no effect on germinal centers [14–15].

NZW/BXSB F1 (W/B) male mice bearing the *Yaa* locus have a duplication of part of the X chromosome that includes the *Tlr7* gene onto the Y chromosome [16–17] and therefore have a 2-fold increase in *Tlr7* expression. Male W/B mice spontaneously develop high titer anti-CL and anti-Sm/RNP autoantibodies that are associated with both anti-phospholipid syndrome and glomerulonephritis whereas females, with only one copy of *Tlr7*, develop a milder disease [18]. Although there are at least 16 genes in the *Yaa* locus, the *Tlr7* duplication is the dominant genetic contributor to the *Yaa* phenotype [7, 19–20]. Furthermore, 4 to 8-fold overexpression of *Tlr7* is sufficient to induce spontaneous onset of SLE in non-autoimmune strains [19].

The purpose of our experiments was to use W/B mice bearing the site-directed anti-CL/DNA autoantibody V_H_ transgene 3H9 [21–22] to determine how an extra *Tlr7* dose influences the selection of naïve and antigen activated autoreactive B cells during the evolution of SLE. We previously showed that 3H9 male NZW/BXSB transgenic mice develop high titer anti-DNA and anti-CL antibodies and develop proteinuria whereas females have a delay in the emergence of autoantibodies and do not become proteinuric. Furthermore although both male and female W/B mice use the 3H9 transgene to encode anti-chromatin and anti-CL antibodies they have differences in selection of the GC repertoire [21]. In these experiments however, there was no competition with non-3H9 B cells and transgenic females did not develop clinical disease. We were therefore not able to control for systemic inflammation or to distinguish the effects of the *Yaa* locus from *Tlr7* duplication on autoantibody production and clinical disease. In the studies reported herein we examined B cell tolerance mechanisms in bone marrow chimeras in which we could study repertoire selection in the setting of physiologic competition with non-autoreactive B cells and evolving inflammation. Our data suggest that an increase in *Tlr7* dose enhances B cell proliferation, selection and diversification in the germinal center.

## Methods

### Mice

C57BL/6 TLR7^-/-^ mice (from Dr Shizuo Akira, University of Osaka) and GFP^+^ C57BL/6 mice (Jackson Laboratories, Bar Harbor, ME) were backcrossed >13 generations to NZW mice and then crossed with 3H9 NZW mice (from Dr. Tony Marion, University of Tennessee). 3H9.GFP^+^.NZW and 3H9.GFP^+^.NZW^TLR7+/-^ females were bred with BXSB males (Jackson Laboratories) to produce GFP^+^ male NZW/BXSB F1 progeny containing either one (W/B^TLR7-/Yaa^) or two (W/B) copies of *Tlr7* with or without the 3H9 transgene (S1 Table). Wild type (wt) NZW/BXSB F1 mice were made by crossing the parental strains (Jackson Labs, Bar Harbor, ME).

Bone marrow chimeras were generated by transfer of 50% 3H9.GFP^+^ and 50% male wild-type (wt) W/B bone marrow into totally irradiated 8–12-wk-old W/B recipients. In the experimental groups, the 3H9.GFP^+^ bone marrow donors were male, male^TLR7-/Yaa^ or female and the bone marrow recipients were universally male. A group of 50% female 3H9.GFP^+^ and 50% female wt donors into female recipients served as non-diseased controls. Groups of control chimeras were made using GFP+ non-3H9 donors (Table 1). Chimeric mice were tested for proteinuria every 2 weeks (Multistick; Fisher, Pittsburgh, PA) and bled periodically for serologic analyses [18, 23]. Mice were sacrificed at the onset of proteinuria 12–20 wk after transplant (Table 1). Studies were approved by the IACUC of the Feinstein Institute for Medical Research (Protocols 2009–054 and 2007–038).

**Table 1:** Donor strains and chimeras.

Chimera Name	Donor 1 NZW/BXSB (GFP+)				Donor 2 NZW/ BXSB	Harvest (Days after transplant)	Recipient NZW/ BXSB
		TLR7	Yaa	3H9			
M WT	M	++	+	-	M	86 +/- 16	M
F WT	F	+	-	-	M	119 +/- 32	M
M 3H9	M	++	+	+	M	95 +/- 31	M
F 3H9	F	+	-	+	M	124 +/- 32	M
M 3H9^TLR7-/Yaa^	M	+	+	+	M	95 +/- 22	M
F 3H9 to F*	F	+	-	+	F	128 +/- 14	F

*Chimeras not proteinuric at time of euthanasia

### Quantitation of autoantibodies to CL and dsDNA

ELISpot assays of spleen cells and serial ELISA assays of serum for antibodies to CL and dsDNA were performed as previously described [18, 24–25]. Reconstituted 3H9/Vκ antibodies were expressed and cotransfected into 293T cells as previously described [21]. Supernatants were normalized to 1ug/ml and tested in serial dilution for binding to CL and dsDNA as above and to histones and chromatin as previously described. These assays were also performed after treatment of the supernatants with DNAse I to remove associated nuclear material present in the cell supernatants [21].

### Flow cytometry and sorting

Spleen and bone marrow B cells were analyzed as previously described [26–28]. For each stain the FITC channel was left open to enumerate GFP^+^ cells.

### Single-cell sorting and PCR

GFP^+^ single cells were sorted from FO, GC and PC B cell subsets from 4–6 mice per group and cDNA preparation and PCR of the 3H9 heavy chain and associated Vκ light chain were performed as previously described [21, 29]. PCR products were sequenced by Genewiz (South Plainfield, NJ), and sequences were identified using the International ImMunoGeneTics (IMGT) database. To distinguish between Vκ5–43*01 and Vκ5–45*01 that differ by three base pairs at the 5’ end and to enumerate somatic mutations, a 5’ leader primers for Vκ5–43/45*01 and Vκ5–48*01 were used to generate full length light chain genes for resequencing. Somatic mutation rates were calculated using a program available on the IMGT website. Similarly, a VH82*01 leader primer was used to generate full length 3H9 heavy chain genes.

### Immunohistochemistry

Spleen tips were incubated in 4% formaldehyde/10% sucrose for 2 hours before freezing in OCT. 7 micron sections were stained with antibodies to IgD and visualized as previously described [30].

### Statistics

**Fig 1:**
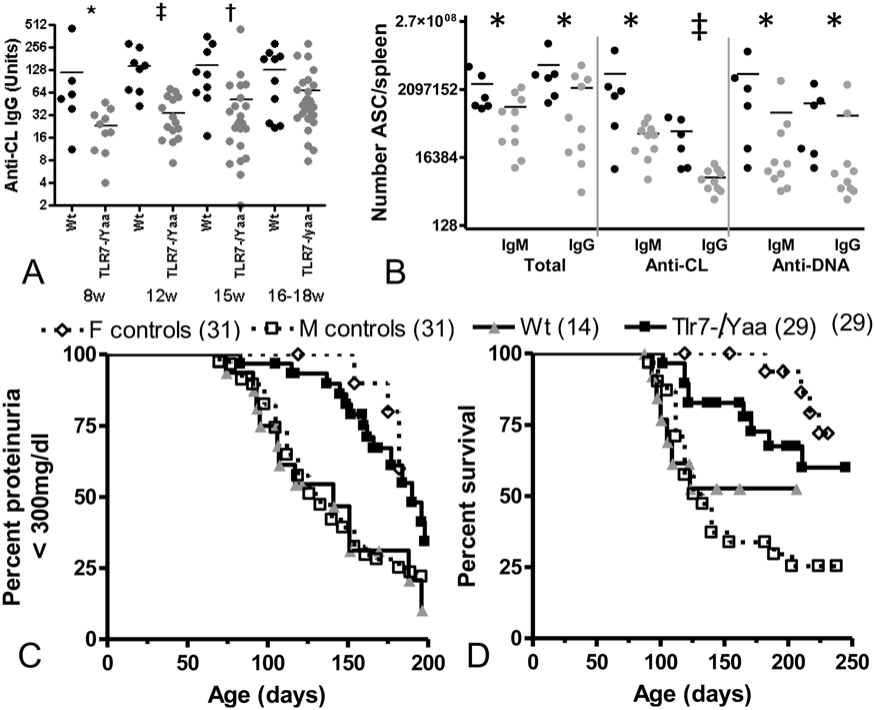
Attenuated disease in NZW/BXSB ^TLR7-/Yaa^ males. A: Titers of IgG anti-CL antibodies were lower in NZW/BXSB^TLR7-/Yaa^ males (grey) compared with their WT littermates (black). B: Autoantibody secreting cells (ASC) were decreased in NZW/BXSB^TLR7-/Yaa^ males (grey) compared with their WT littermates (black). B, C: NZW/BXSB^TLR7-/Yaa^ males had a delay in onset of proteinuria (C) and an improved survival (D) compared with their WT littermates or historical male controls. * p < 0.05, † p < 0.01, ‡ p<.001.

**Fig 2:**
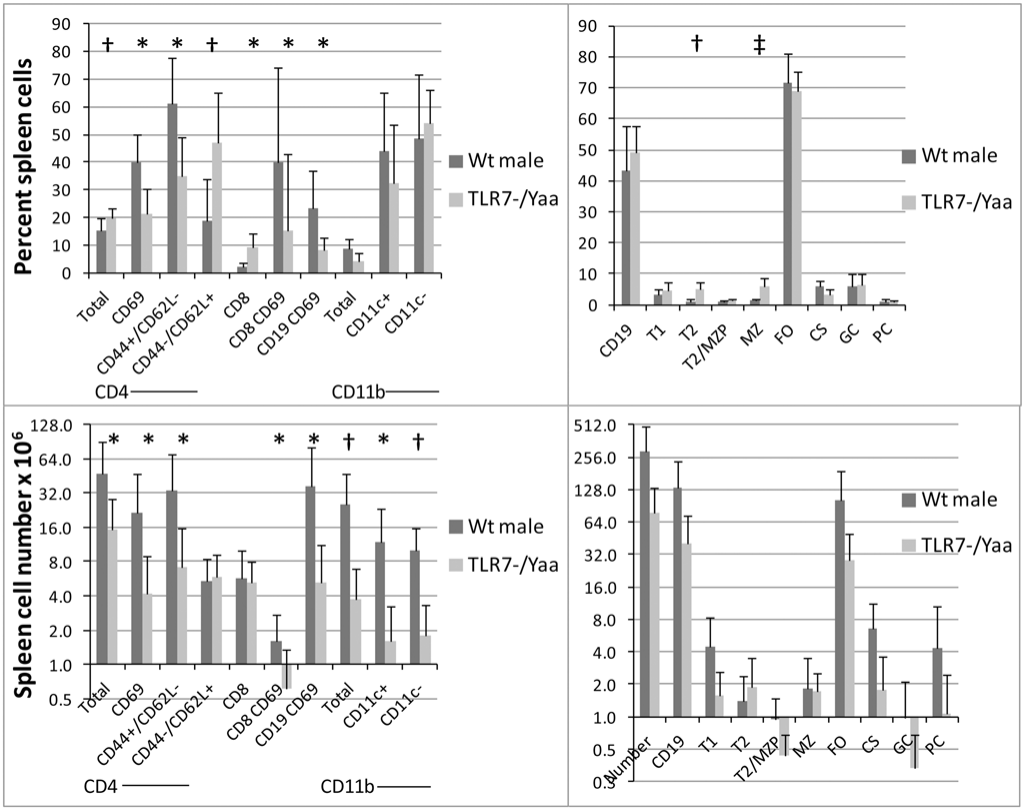
Spleen cell phenotype in NZW/BXSB^TLR7-/Yaa^ mice (n = 10) and WT littermates (n = 5): A: T cell, B cell and dendritic cell percentages. CD4, CD8, CD19 and CD11B subsets are shown as a percentage of the parental cell type. B: Total spleen cell numbers of T cells, B cells and dendritic cell total numbers. Note the decrease in activated (CD69^+^) and effector/memory (CD44^+^/CD62L^-^) CD4 T cells, activated (CD69^+^) B cells and dendritic cells in the TLR7^-/Yaa^ mice. C: B cell subsets as a percentage of spleen lymphocytes. D: Total B cell numbers per spleen. Note the decrease in follicular and class switched B cells and the preservation of T2 cells in the NZW/BXSB^TLR7-/Yaa^ mice. * p < 0.05, † p < 0.01.

**Fig 3:**
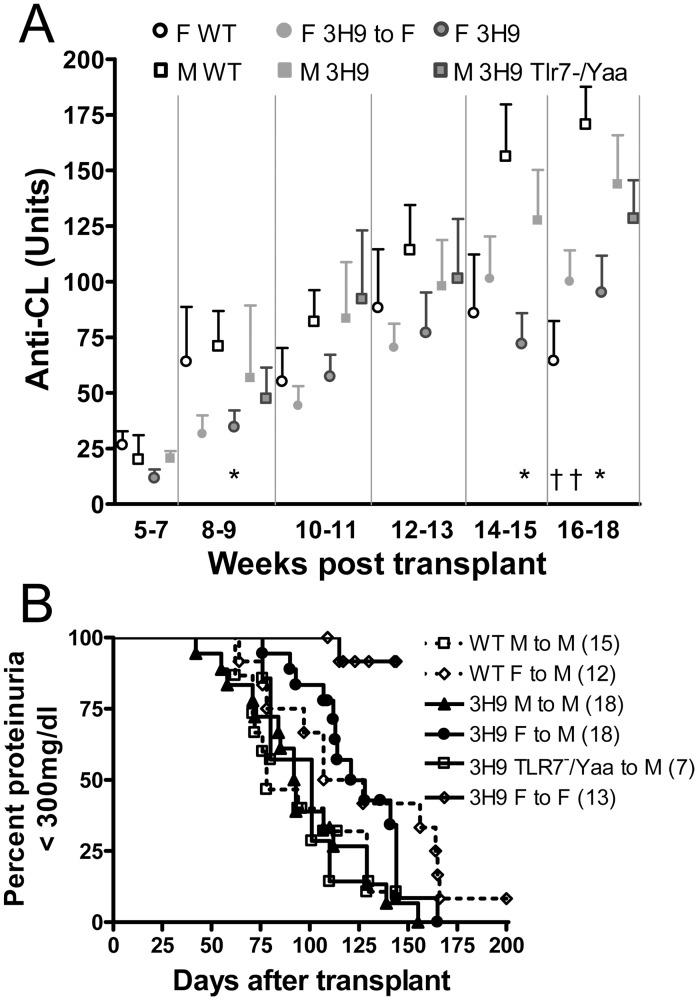
Clinical outcome of the chimeras: A: Serum levels of anti-CL antibodies (mean + SEM) in the female (circles) and male (squares) chimeras. Antibody titers increased with age in all the chimeras. Levels of anti-CL antibodies were lower in the female chimeras by age 16–18 weeks. Comparisons are with male WT controls. * p < 0.05, † p < 0.01. B: Time to fixed proteinuria onset. Note the marked delay in the 3H9 F to F chimeras (p < 0.0001 vs. males) and the modest delay in the 3H9 F to M chimeras (p < 0.0025 vs. males).

**Fig 4:**
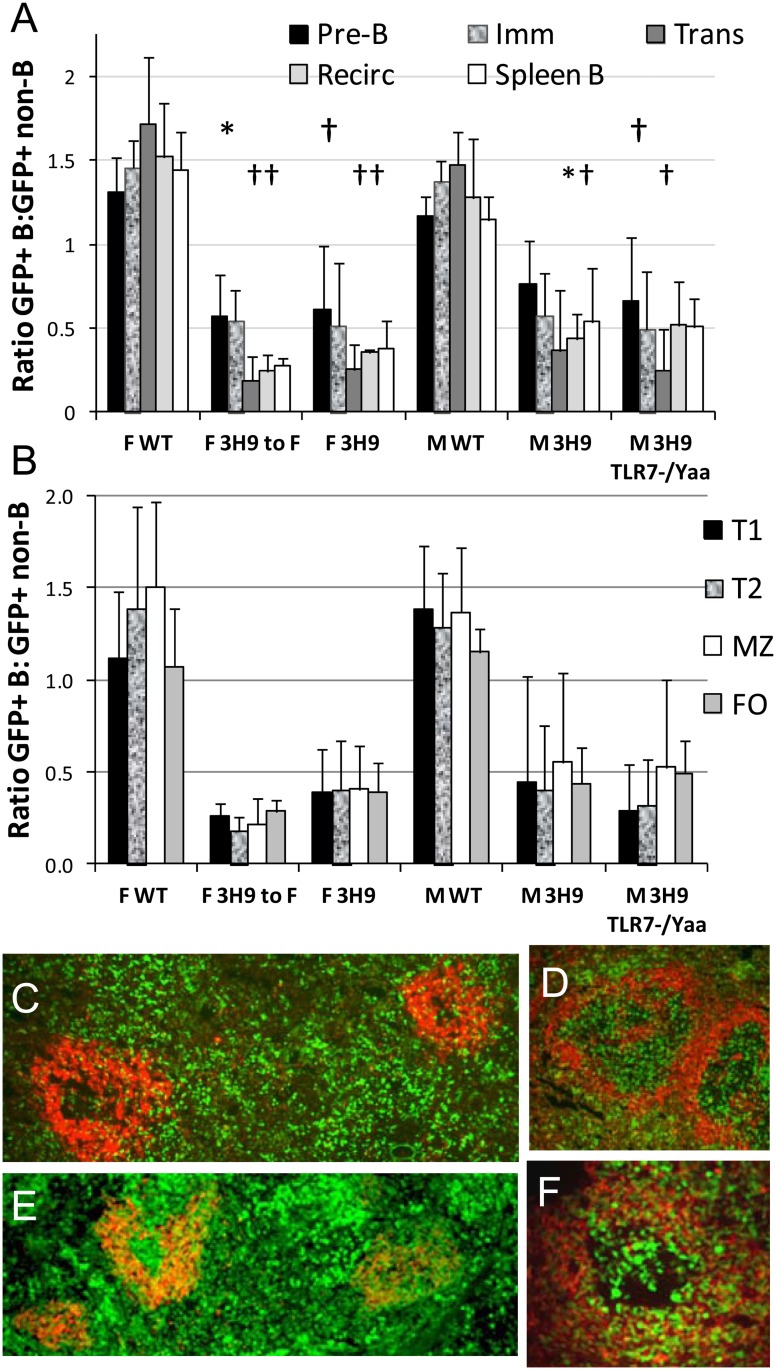
Deletion of 3H9 B cells occurs in the bone marrow. A. Ratio of GFP^+^ B cells: GFP^+^ B220^-^ non B cells in bone marrow subsets shows deletion occurring at the pre-B stage in the 3H9 chimeras (upper symbols show comparisons of pre-B cell ratios with those of WT female or male counterparts). Further deletion occurred between the pre-B and transitional (Trans) and recirculating (Recirc) B cells (lower row of symbols). n = 5–12 per group, * p < 0.025, † p < 0.01. B. Ratio of GFP^+^ B cells: GFP^+^ T cells in spleens shows no further deletion between the T1 and marginal zone (MZ) or follicular (FO) stages. C-F. Representative spleens from 3H9 M chimeras (C, D) and WT M chimeras (E,F) stained with anti-IgD (red). Note the exclusion of GFP^+^ cells from the B cell zones of the follicles in the chimeric mice. Magnification 10X (C,E) or 40X (D,F).

**Fig 5:**
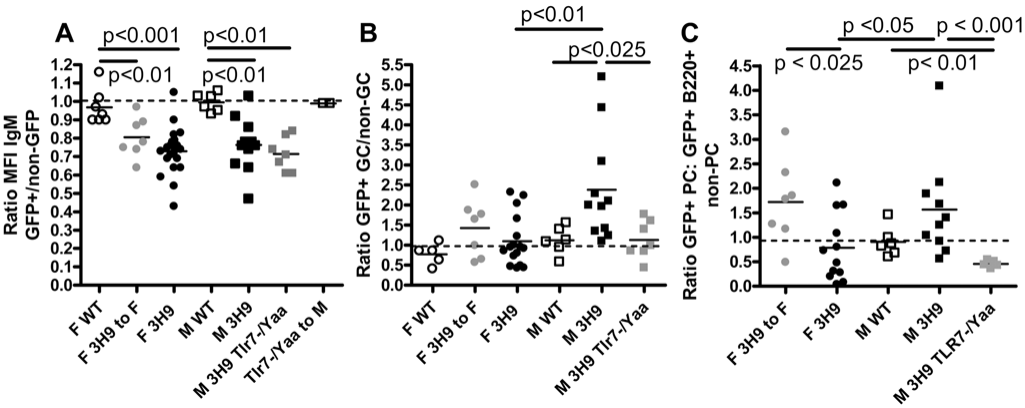
Downregulation of surface IgM on 3H9 B cells does not prevent GC entry. A. Downregulation of sIgM as assessed by the ratio of sIgM on GFP^+^ follicular B cells: sIgM on GFP^-^ follicular B cells occurs in all the 3H9 chimeras regardless of gender or TLR7 status. B. Overrepresentation of 3H9 B cells among GC B cells in M 3H9 chimeras as assessed by the ratio of GFP^+^ GC cells: GFP^-^ GC cells. C. Underrepresentation 3H9 B cells among PC B cells from F 3H9 and M 3H9^Tlr7-/Yaa^ chimeras as assessed by the ratio of GFP^+^ PC cells: GFP+ B220+ non-PC cells.

**Fig 6:**
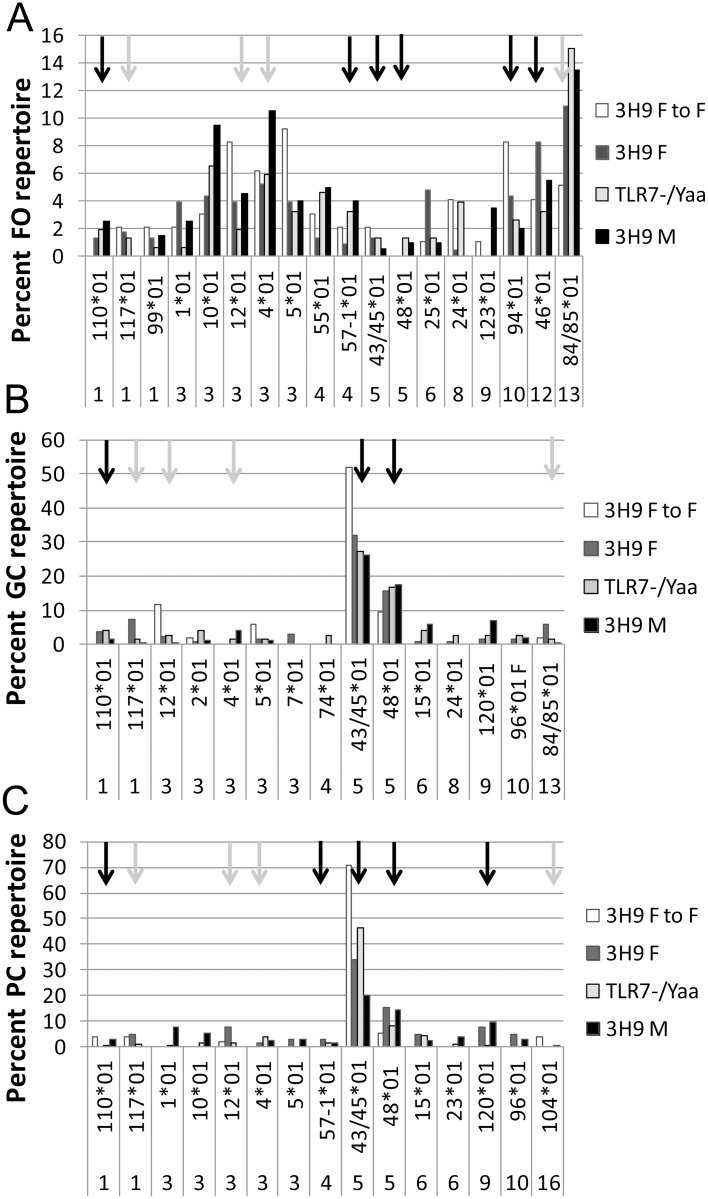
Repertoire analysis of 3H9-associated V_κ_ chains in follicular (A) germinal center (B) and plasma cell (C) subsets of chimeric mice. Genes that were in the top 10 contributors to the χ^2^ value and constitute >2.5% of the repertoire in any of the comparisons are shown. 55–200 sequences from 3–6 mice per group were analyzed per subset. The y axis shows percent of the total repertoire for each subset. Black arrows indicate that the reconstituted 3H9/Vκ encodes an autoreactive antibody. Grey arrows indicate that the reconstituted 3H9/Vκ encodes a non-autoreactive antibody. The complete dataset is shown in S2 Table.

**Fig 7:**
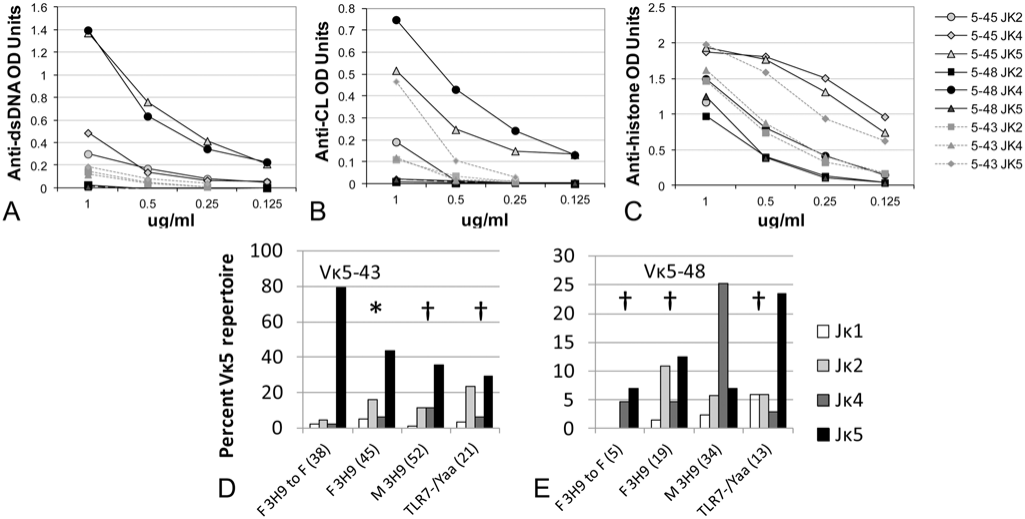
Binding characteristics of Vκ5 encoded light chains vary with Jκ usage. Serial dilutions of each heavy and light chain pair were tested for the reactivity with dsDNA (A), CL (B) or histones (C).Distribution of Jκ regions among Vκ5–45 (D) and Vκ5–48 (E) encoded light chains as a percentage of total Vκ5 encoded light chains is shown. Comparisons are with F 3H9 to F chimeras for Vκ5–45 and with M3H9 chimeras for Vκ5–48. * p < 0.05, † p < 0.01.

Proteinuria and survival data were analyzed using Kaplan-Meier curves and log rank test. Comparisons shown in Figs. 1A, 2 and 3A were performed using Mann-Whitney test. *p* values ≤ 0.05 were considered significant. Comparisons in Fig. 4 were performed using pair-wise Mann-Whitney test with a Bonferroni adjustment to account for multiple testing. In order to preserve an overall 5% significance level, a pairwise comparison was considered statistically significant if p < 0.025. For Fig. 5 the Kruskal-Wallis test was used to compare the M and F wt with their respective experimental groups. Upon finding a significant difference, Bonferroni adjusted multiple pairwise comparisons of the WT group to each of the other two gender-matched groups, were carried out using Mann-Whitney tests. A *p* value of <0.025 was considered significant. For all male vs. female comparisons the Mann-Whitney test was used. Statistical analysis of the Vκ repertoire data shown in Fig. 6 was performed as previously described [31]. Owing to the typically small sample sizes of single cells, the usual Pearson χ^2^ statistic is not valid for determining whether differences exist in the distributions of L chains across B cell subsets. Instead, the Fisher exact test is applicable. However, the computing time required for running the exact test on large, sparse tables is prohibitive and, moreover, the power of the test is low. Accordingly, identifying L chains of interest without using formal inferential methods (i.e., without using *p* values or confidence intervals) is needed. This exploratory approach is frequently used in identifying interesting genes in gene expression microarray studies [32–34]. To this end, we used a novel method for “screening” the *r* × *c* table for cell frequencies that appear to be inconsistent with the null hypothesis that the B cell subset and L chain distributions are independent. This method uses a graphical approach (scree plots) to finding the entries in the table that depart most from the null hypothesis. For additional details on the statistical methodology please refer to Lesser *et al* [35]. Comparisons in Fig. 7 were performed using chi-square analysis. Mutation frequencies in each subset were compared using the Mann-Whitney test.

## Results

### Phenotype of NZW/BXSB^TLR7-.Yaa^ mice

The phenotype of male NZW/BXSB^TLR7-/Yaa^ mice has not been reported and was compared with that of wt male littermates and with historical male and female W/B controls. NZW/BXSB^TLR7-/Yaa^ mice had lower titers of anti-CL antibodies than wt controls up until the age of 16 weeks (Fig. 1A). Anti-dsDNA antibody titers appeared after 12 weeks of age in the wt mice and trended lower in the NZW/BXSB^TLR7-/Yaa^ mice after the age of 15 weeks (p = 0.07, not shown). Only low titers of anti-Sm/RNP antibodies were present by 18 weeks of age with a trend towards lower titers in NZW/BXSB^TLR7-/Yaa^ mice (p = 0.09, not shown). Fewer antibody and autoantibody secreting B cells, particularly IgG anti-CL producing B cells were present in the spleens of NZW/BXSB^TLR7-/Yaa^ mice harvested at 18–22 weeks of age, compared with their age matched wt littermates (Fig. 1B). These data show that TLR7 dose regulates the anti-CL response in wt NZW/BXSB males. Proteinuria onset was significantly delayed in NZW/BXSB^TLR7-/Yaa^ mice compared with wt littermates (Fig. 1C, p = 0.011) and this was associated with a decrease in their mortality (Fig. 1D, p = 0.05). Proteinuria onset and survival were indistinguishable between wt littermates and historical male controls [18]. Survival was modestly decreased in NZW/BXSB^TLR7-/Yaa^ mice compared with historical female controls [23] but this difference did not reach statistical significance.

The spleens of NZW/BXSB^TLR7-/Yaa^ mice harvested between 18–22 weeks of age were smaller than those of their wt littermates and contained fewer total B cells, dendritic cells and activated B and T cells reflecting a decreased overall inflammatory burden (Fig. 2A, B). NZW/BXSB^TLR7-/Yaa^ mice displayed a normal T1:T2 ratio and normal percentage of marginal zone B cells. By contrast wt mice manifested a reversed T1:T2 ratio (p = 0.05) and had a decrease in frequency (p = 0.0007) but not the total number of marginal zone B cells. In accordance with the serologic data, NZW/BXSB^TLR7-/Yaa^ mice had lower numbers of class switched B cells than their wt littermates (p = 0.05) and a trend towards a decrease in plasma cells (p = 0.09, Fig. 2C, D).

### Phenotype of 3H9 W/B bone marrow chimeras

Male 3H9 (M 3H9), male 3H9^TLR7-/Yaa^ (M 3H9^TLR7-/Yaa^), female 3H9 (F 3H9), male wt (M WT) mice, and female wt (F WT) chimeric mice (Table 1) developed IgG autoantibodies to CL by 10–13 weeks post-transplant. By16–18 weeks, titers of anti-CL antibodies were lower in female chimeras than in M WT chimeras regardless of 3H9 status but there was no difference between M WT and either M 3H9 or M 3H9^TLR7-/Yaa^ chimeras (Fig. 3A). Development of high titer IgG anti-DNA antibodies was sporadic in each group, with a significant increase in titers at 14–15 weeks post-transplant only in the M WT chimeras (data not shown).

Proteinuria was markedly delayed in the F 3H9 to F chimeric mice (p < 0.0001 vs. males) and developed with a modest delay in the female to male compared to the male chimera sets regardless of 3H9 status (p = 0.0025 M vs. F). M 3H9^TLR7-/Yaa^ chimeras developed proteinuria with similar kinetics to the male controls (Fig. 3B, Table 1). These data, in sum, suggest that the *Yaa* locus confers a slight disadvantage beyond the major effect conferred by the extra copy of *Tlr7* in NZW/BXSB males.

### B cell phenotype of W/B bone marrow chimeric mice

Phenotypic analysis of spleen B cells at the time of sacrifice indicated that F 3H9 to F chimeras had a higher frequency of MZ B cells and a lower frequency of FO B cells and plasma cells than all the other chimeras (S1A Fig.). This is consistent with disease protection in fully female mice. The spleens of these mice were also significantly smaller than those of the other chimeras (52.3 +/- 26.8 x 10^6^ cells compared with 149.4 +/-78.7 x 10^6^ cells in M 3H9 chimeras, p <0.0001, and 126.3 +/-55.3 x 10^6^ cells in F 3H9 chimeras, p < 0.01) such that the number of FO B cells, GC B cells and plasma cells was significantly less. All the other chimeras phenotypically resembled M WT (S1B Fig. and data not shown).

As a measure of the selection pressure against transgenic B cells we assessed deletion of 3H9 B cells in each subset by calculating the ratio of % GFP^+^ B cells to % GFP^+^ non-B cells (BM non-B cells for BM and average of spleen CD4 and CD8 cells for spleens). In the absence of deletion this ratio should be 1. Ratios of % GFP^+^ CD4 cells to % GFP^+^ CD8 or CD11b+ cells were close to 1 for all chimeras (not shown) indicating no selection pressure against these cell types based on their TLR7 dose. By contrast, all the 3H9 chimeras exhibited deletion of GFP^+^/3H9^+^ transgenic B cells in the bone marrow. This was already evident at the pre-B cell stage (Fig. 4A) with further deletion occurring between the pre-B cell and transitional stages in the BM. No further deletion occurred between the transitional and recirculating or between the peripheral T1 and FO stages (Fig. 4B). Thus, central deletion is the major contributor to tolerance induction of 3H9 B cells in W/B mice. Furthermore, deletion of 3H9+ cells was more stringent in 3H9 F to F chimeras than in the male chimeras (spleen CD19^+^/non-B ratio 0.28 +/- 0.04 vs. 0.53 +/- 0.17 in M 3H9, p = 0.01; 0.51 +/- 0.17 in M 3H9^TLR7-/Yaa^, p < 0.05) with an intermediate phenotype of the F 3H9 chimeras (Fig. 4B). By contrast, GFP^+^ B cells were not deleted in the BM when the donors were 3H9 negative. These data were confirmed by immunohistochemical studies that showed follicular exclusion of GFP^+^ cells in the 3H9 chimeras but not in the WT chimeras (Fig. 4C-F).

Surface IgM staining was decreased on splenic GFP^+^ follicular cells from 3H9 chimeric mice compared to GFP^-^ follicular B cells from the same mice or to non-3H9 GFP^+^ cells from M or F wt chimeras, suggesting that the remaining 3H9^+^ cells have encountered autoantigen in the periphery [36–37] (Fig. 5A). This BCR downregulation did not however prevent 3H9^+^ cells from entering the GCs or appearing in the plasma cell compartment in any of the chimeras. Importantly, GFP^+^ transgenic B cells were preferentially enriched in the GCs of M 3H9 compared to F 3H9 and M 3H9^TLR7-/Yaa^ chimeric mice (Fig. 5B). This suggests an effect of TLR7 dose on GC selection and/or proliferation of autoreactive B cells. A similar effect was observed in the plasma cell compartment (Fig. 5C).

### Single-cell PCR analysis of the transgenic 3H9 B cell light chain repertoire

To determine whether *Tlr7* dose and/or the *Yaa* locus influenced selection of the naïve or antigen selected repertoires we performed single cell PCR of light chains associated with the 3H9 heavy chain in selected B cell subsets. We have previously established that reconstituted unmutated 3H9/Vκ combinations using Vκ1–117, 3–4, 3–12, 13–85, 9–120 and 16–104 are non-reactive with dsDNA, CL, histones or chromatin whereas Vκ1–110, 4–57, 5–43, 5–45, 5–48, 10–94 and 12–46 react with one or more of these antigens ([21] and data not shown). Analysis of the follicular light chain repertoire revealed several differences between F to F 3H9, F 3H9, M 3H9 and M 3H9^TLR7-.Yaa^ follicular B cells. However there was no clear effect of either *Yaa* status or *Tlr7* dose on selection against known lupus related autoreactive specificities in the follicular repertoire (Fig. 6A, S2, S3 Tables).

The repertoire of light chains associated with 3H9+/IgG GC B cells was highly restricted in all four sets of chimeric mice, with overrepresentation of Vκ5–43/45 and Vκ5–48 as previously observed in the parental 3H9 W/B strain [21] (Fig. 6B, S2, S3 Tables). Among the Vκ5 encoded light chains, Vκ5–43/45 was over represented in the F 3H9 to F chimeras compared with all the other chimeras (p <0.01 vs. the male chimeras and p<0.05 vs. F 3H9 chimeras). V3–5*01 and Vκ3–12*01 were also over represented in the F 3H9 to F chimeras. The 3H9+ plasma cell repertoire was similar to that of the GC repertoire. M 3H9 GC B cells and plasma cells exhibited the most light chain diversity, with a decrease in expression of Vκ5–43/45*01 light chains and more frequent clonal expansions of other light chains (Fig. 6C, S2, S3 Tables).

We have previously shown that the autoreactive specificity of germline encoded 3H9/Vκ5 antibodies in non-chimeric 3H9 W/B males and females is influenced by the associated Jκ. In these studies there was preferential selection of Vκ5–43/Jκ5 and Vκ5–48/Jκ4 in 3H9 W/B males and of Vκ5–43/Jκ2, Vκ5–43/Jκ4 and Vκ5–48/Jκ5 in 3H9 W/B females [21]. Analysis of the antigenic specificity of these H/L combinations showed that 3H9/Vκ5–48/Jκ4 had the highest affinity for CL and dsDNA whereas 3H9/Vκ5–43/Jκ5 bound to histone and dsDNA (Fig. 7A-C). Some of this reactivity was attenuated after DNAse treatment and Protein A column chromatography but 3H9/Vκ5–43/Jκ5 and 3H9/Vκ5–48/Jκ4 still had higher affinity for chromatin than the other Vκ5–43/Jκ5 and Vκ5–48/Jκ4 H/L combinations [21]. Surprisingly, the 3H9/Vκ5–43/45 Jκ5 pair was most frequent in the F 3H9 to F chimeras and least frequent in the 3H9 male chimeras (Fig. 7D). Vκ5–43 and Vκ5–45 have only 2 amino acid differences (S to R in FR1 and N to Y in CDR1) and can only be distinguished by full length sequencing of the light chain. 3H9/Vκ5–45/Jk5 binds with higher affinity to dsDNA, CL and histones than does 3H9/Vκ5–43/Jκ5 (Fig. 7A-C) and retains specificity for dsDNA and CL after DNAse 1 treatment and purification [21]. We therefore performed full length sequencing of Vκ5–43/45 light chains from the different chimeras. We found that Vκ5–45 constituted 22% of the Vκ5–43/45 light chains in the GCs of M 3H9 chimeras compared with 12–13% in F 3H9 and M 3H9^TLR7-/Yaa^ chimeric mice and 17% in F 3H9 to F chimeric mice; these differences were not significantly different. Similarly, Vκ5–45 was represented equivalently in the plasma cell repertoire of all chimeras.

3H9/Vκ5–48/Jκ4, that encodes for anti-CL activity accounted for 25.3% of the Vκ5 encoded light chain repertoire in the GCs of M 3H9 chimeras compared with < 5% in the other 3H9 chimeras (Fig. 7E, p < 0.01 compared with F 3H9 chimeras and p < 0.001 compared with M 3H9^TLR7-/Yaa^ chimeras). These data in sum show selection against Vκ5–43/Jκ5 and selection for Vκ5–48/Jκ4 in the 3H9 male chimeras, with the opposite phenotype in the 3H9 F to F chimeras and an intermediate phenotype in the other chimeras. In addition the 3H9-associated light chain repertoire in the 3H9 male chimeras exhibits more diversity than that of the other chimeras.

### Analysis of somatic mutations

We have previously shown higher affinity CL and dsDNA binding activity of 3H9/Vκ5–43 and 3H9/Vκ5–48 pairs can be acquired as a result of somatic mutations [21]. Since Vκ5–43 and Vκ5–48 were the dominant light chains used in association with class switched 3H9 B cells in the GCs in all the chimeras we enumerated the number of mutations in the FR regions and in CDRs of full length Vκ5–43 sequences from M 3H9, F 3H9 3H9^TLR7-/Yaa^ and F 3H9 to F chimeras and in full length Vκ5–48 light chains from M 3H9 (18) and F 3H9 (19) chimeras. Light chain Vκ5–43 sequences from the GC B cells of F 3H9 chimeras had significantly fewer mutations than those from M 3H9 chimeras (average 0.9 vs. 3.0 mutations per sequence p< 0.05—Table 2); this difference was not however observed in the Vκ5–48 sequences (data not shown) or in Vκ5–43 encoded sequences from plasma cells (Table 2).

**Table 2:** Mutation analysis of 3H9-associated Vκ5.43 light chains.

				Framework		CDR	
Subset	Chimera	Number sequences	Mutations/Seq	Silent	Replacement	Silent	Replacement
GC	3H9 M	28	3.0	45	15	13	12
	3H9 F	29	0.9*	14	8	2	3
	3H9 ^TLR7-/Yaa^	13	1.9	5	13	1	6
	3H9 F to F	16	8.5*	24	54	8	41
Plasma	3H9 M	25	3	10	27	6	5
	3H9 F	13	5.9†	17	35	3	22
	3H9 ^TLR7-/Yaa^	54	3.63	34	95	28	39
	3H9 F to F	19	8*	37	52	14	33

*p <0.001 compared with 3H9 M

†p < 0.05 compared with 3H9 M

Surprisingly, the Vκ5–43 encoded light chains in the 3H9 F to F GC B cells and plasma cells had significantly more mutations than those of the other chimeras ((p < 0.001—Table 2). The light chain CDR1 makes an important contribution to antigen binding, with arginine, asparagine and lysine having DNA and chromatin binding capacity [38–40]. For Vκ5–43 the CDR1 consists of an asparagine rich sequence QNISNN. In F 3H9 to F chimeras one or both of the two terminal asparagines was replaced in 13/18 GC sequences and 11/20 plasma cell sequences, mostly by serine or isoleucine. By contrast, replacement of these asparagines occurred in only 1/29 GC sequences from F 3H9 (p = 0.0001), 0/28 GC sequences from M 3H9 (p < 0.0001) and 2/16 GC sequences from M 3H9^TLR7-/Yaa^ mice (p < 0.001). The two terminal asparagines in CDR1 were also conserved in 10/11 Vκ5–43 encoded light chains from anti-DNA/anti-CL hybridomas derived from male 3H9 parental mice in which there was an average of 10 mutations per sequence (p = 0.002) [21]. The heavy chain CDR2 of 3H9 has a mutation from G to R and the CDR3 has two arginine residues that contribute to the autoreactive specificity of 3H9 encoded antibodies [38–40]. Of the 110 heavy chains recovered from wells expressing Vκ5–43/45 light chains, representing all the chimeras, the CDR2 arginine was conserved in 108 and the two CDR3 arginines were conserved in 108 with the other two manifesting a conservative R to K mutation.

## Discussion

The purpose of our experiments was to use W/B mice bearing the site directed anti-CL/anti-DNA autoantibody V_H_ transgene 3H9 to determine how *Tlr7* expression and the *Yaa* locus influence the selection of naïve and antigen activated autoreactive B cells during the evolution of SLE. 60% of L chains that associate with 3H9 are permissive for autoreactivity to DNA or CL [39]. Tolerance in non-autoimmune 3H9 mice is maintained by receptor editing of the light and, to a lesser extent, the heavy chains to yield a less autoreactive naive repertoire [22, 39, 41–42], by follicular exclusion of autoreactive B cells [43], and by negative selection of autoreactive B cells in the GCs [44]. By contrast, 3H9 encoded autoantibodies using a variety of light chains are detected among autoreactive IgG hybridomas from 3H9 transgenic lupus-prone MRL/lpr mice [45]. TLR7 is required for the anti-RNA response and the absence of TLR signaling significantly attenuates the anti-CL response [46]. NZW/BXSB^TLR7-/Yaa^ mice had significantly lower anti-CL titers and a decreased number of anti-CL AFCs compared with their WT counterparts. Thus *Tlr7* dose significantly influences the anti-CL response in NZW/BXSB mice. Because 3H9 does not encode for anti-RNA antibodies we focused on the anti-CL response in these studies.

The role of B cell intrinsic TLR7 in regulating class switched autoantibody production and renal inflammation has been studied in mice with B cell specific genetic deficiency of *Tlr7* or its adaptor *MyD88*. B cell depletion of *MyD88* greatly reduces titers of IgG antinuclear antibodies and the GC B cell population fails to expand with age [47–48] resulting in a decrease in renal immune complex deposition and renal damage. Similarly, in mice with *Tlr7* deficient B cells anti-RNA responses are abrogated and renal damage is decreased [14]. In *Unc93b1* deficient MRL/lpr mice that have no signaling through any nucleic acid recognizing TLRs, all antinuclear antibodies are completely abrogated. When both WT and Unc93b1 deficient B cells are co-transferred into B6/lpr mice autoantibodies are preferentially made by the WT cells [46]. Finally, in *Tlr7* deficient Sle1 mice, the presence of spontaneous germinal centers and autoantibody production are dependent on B cell intrinsic *Tlr7* (15). These data all point to an intrinsic B cell function of TLR7 in promoting autoantibody production and subsequent tissue damage. Our analysis of the effect of the effect of excess *Tlr7* dose on GC selection of H/L pairs with known antigenic specificity is consistent with this concept and yielded several important mechanistic findings.

An important design aspect of this study was to provide an environment in which immature, 3H9 positive B cells harboring *Yaa* or expressing one or two copies of *Tlr*7 were subject to competition with wt non-3H9 cells for survival; this allows deletional tolerance mechanisms to be operative. In addition the transgenic B cells were exposed to an inflammatory environment as disease evolved. The GFP marker allowed us to sort single transgenic cells from each B cell subset even though they represented only a small percentage of the B cell repertoire.

We first showed that the major site for deletion of 3H9 B cells is central with little further deletion occurring in the periphery. This is in contrast to the autoreactive anti-dsDNA D42 transgenic system in which deletion of autoreactive specificities occurs predominantly at the transitional stage in the periphery [31]. Autoreactivity of the remaining 3H9 B cells appeared to persist in the periphery as manifested by downregulation of surface IgM on the mature naïve transgenic cells. Downregulation of surface BCR in non-anergic autoreactive B cells has been postulated to be a mechanism for preserving diversity of the B cell repertoire and perhaps allowing subsequent mutation and selection away from autoreactivity during antigen specific responses [37, 49].

Examination of the light chains associated with 3H9 in follicular B cells revealed several differences between the various chimeras but it was not possible to distinguish a clear pattern of selection against autoreactivity. Importantly however, the most commonly used autoreactive heavy/light chain pairs in the follicular repertoire 3H9/10–94*01 and 3H9/12–46*01/Jκ2 were excluded from the GC and plasma cell repertoires. Whether these autoreactive B cells are segregated into the follicular subset with the lowest sIgM expression will need further study.

We next demonstrated that male W/B 3H9 B cells bearing 2 copies of *Tlr7* are preferentially expanded in the GC compared to W/B female or male W/B^TLR7-/Yaa^ 3H9 B cells with only 1 copy of *Tlr7*. To further dissect the effects of TLR7 and *Yaa* on GC selection of 3H9+ B cells we examined the repertoire of light chains associated with the 3H9 heavy chain in class switched GC B cells and plasma cells. We have previously shown by single cell PCR analysis and analysis of hybridomas that Vκ5–43 and Vκ5–48 light chains are overrepresented in the GC repertoire of both male and female 3H9 W/B mice but that the preferred VJ combinations differ between males and females. Higher affinity autoreactive B cells expressing Vκ 5–43/Jκ5 and Vκ 5–48/Jκ4 are preferentially selected into and expanded in the GCs of male 3H9 W/B mice compared with females. This suggests that selection of autoreactive B cells into the GC may be partially dependent on TLR7 [21]. We show here that when interclonal competition for GC entry is provided, all the chimeras select the Vκ5–43/Jκ5 encoded light chain that confers anti-chromatin activity; this specificity has been shown not to be influenced by *Tlr7* dose [5]. However the repertoire of M 3H9 chimeric mice diversifies away from Vκ5–43/Jκ5 and demonstrates preferential use of Vκ5–48/Jκ4 that confers anti-CL reactivity. Overall, these data support the notion that TLR7 over-expression influences the stringency of GC selection and facilitates clonal expansion of autoreactive B cells in the GC.

Somatic mutations are linked to proliferation and this occurs in the germinal center dark zone [13] that is smaller when TLR7 is absent [50]. We therefore examined the effect of TLR7 overexpression on the frequency of somatic mutation in GC B cells. We were unable to demonstrate a consistent effect of either *Yaa* or *Tlr7* dose on the frequency of somatic mutations in Vκ5 encoded light chains. It was intriguing however, that in GC B cells the rate of somatic mutations was low in 3H9 associated Vκ5–43 encoded light chains from F 3H9 chimeras in which non-*Yaa* B cells compete with male *Yaa* B cells for survival and GC entry. Surprisingly, there was a high frequency of somatic mutations in the analogous GC and plasma cell populations from 3H9 F to F chimeras in which there was no competition between *Yaa* and non-*Yaa* B cells. In the latter mice, B cells encoding the autoreactive 3H9/Vκ5–43/Jκ5 pair could presumably compete equally for T cell help, whereas in the former we postulate that the same cells may not compete as well for the signals that facilitate dark zone entry. Nevertheless, the total number of spleen cells, GCs and plasma cells was substantially lower in the 3H9 F to F chimeras than in the other chimeras. This most likely reflects additional effects of *Tlr7* dose either on the antigen presenting ability of B cells or on the myeloid cells that facilitate T cell expansion and inflammation [47–48].

Previous studies have shown that downregulation of the BCR in autoreactive B cells does not prevent GC B cell expansion of these cells but may influence maturation to plasma cells [36]. In addition TLR7 has been shown to influence the survival of plasma cells in an anti-viral response [50]. We found a modest decrease in the ratio of GFP+ plasma cells compared with GFP+ B220+ cells in the F 3H9 and M 3H9^TLR7-/Yaa^ compared with the M 3H9 chimeras. In addition, there were differences in the spleen plasma cell repertoire between the chimeras similar to those of the GC repertoire, with a highly restricted light chain repertoire in the F 3H9 to F controls and the most light chain diversity in the 3H9 M chimeras.

The presence of at least one *Tlr7* allele has previously been shown to be necessary for an optimal GC response to viral infection and for spontaneous GC activation in lupus prone mice. Our findings in sum show that in an inflammatory microenvironment TLR7 overexpression contributes significantly to the loss of B cell tolerance in the GCs of *Yaa* bearing lupus prone mice where it influences selection, proliferation and diversification of the repertoire of autoreactive B cells in the GCs.

## Supporting Information

S1 Fig Percent (A) and number (B) of spleen B cell subsets in the chimeras. The 3H9 F to F chimeras have a higher percentage of marginal zone (MZ) B cells and a lower total number of follicular (FO), class switched (CS), germinal center (GC) and plasma cells (PC) than all the other chimeras. Comparisons are with male WT chimeras. n = 5–12 per group. * p < 0.05, † p < 0.01.
(TIF)

S1 Table Experimental mouse strains. Generation of mouse strains used for analysis of the TLR7^-/Yaa^ phenotype and as donors for the bone marrow chimeras.
(DOCX)

S2 Table Pairwise comparisons of Vκ repertoires of FO, GC and PC subsets. The Table shows the number of Vκ genes represented in each comparison, the number of genes that contribute the top 50% of the statistical difference in the **λ** ^**2**^ analysis and the percent contribution of the most differentially expressed gene. Individual genes that contribute >5% of the statistical difference in the **λ** ^**2**^ analysis are shown on the right for each comparison. See methods section for a description of the statistical analysis.
(DOCX)

S3 Table 3H9 associated Vκ usage of B cell subsets from chimeric mice. Vκ usage of each 3H9-expressing single cells sorted from each of the follicular, germinal center and plasma cell subsets of bone marrow chimeric mice. The number of cells expressing each Vκ is shown for each set of chimeras. See Fig. 6 for graphical representation of selected genes.
(DOCX)

## References

[1] Wardemann H, Nussenzweig MC. B-cell self-tolerance in humans. Adv Immunol. 2007;95:83–110. 17869611 10.1016/S0065-2776(07)95003-8

[2] Vuyyuru R, Mohan C, Manser T, Rahman ZS. The lupus susceptibility locus Sle1 breaches peripheral B cell tolerance at the antibody-forming cell and germinal center checkpoints. J Immunol. 2009;183:5716–27. 10.4049/jimmunol.0804215 19828626 PMC2879885

[3] Pugh-Bernard AE, Silverman GJ, Cappione AJ, Villano ME, Ryan DH, Insel RA, et al Regulation of inherently autoreactive VH4–34 B cells in the maintenance of human B cell tolerance. J Clin Invest. 2001;108:1061–70. 11581307 10.1172/JCI12462 PMC200949

[4] Christensen SR, Shupe J, Nickerson K, Kashgarian M, Flavell RA, Shlomchik MJ. Toll-like receptor 7 and TLR9 dictate autoantibody specificity and have opposing inflammatory and regulatory roles in a murine model of lupus. Immunity. 2006;25:417–28. 16973389 10.1016/j.immuni.2006.07.013

[5] Hwang SH, Lee H, Yamamoto M, Jones LA, Dayalan J, Hopkins R, et al B cell TLR7 expression drives anti-RNA autoantibody production and exacerbates disease in systemic lupus erythematosus-prone mice. J Immunol. 2012;189:5786–96. 10.4049/jimmunol.1202195 23150717 PMC3544945

[6] Fossati L, Sobel ES, Iwamoto M, Cohen PL, Eisenberg RA, Izui S. The Yaa gene-mediated acceleration of murine lupus: Yaa- T cells from non-autoimmune mice collaborate with Yaa+ B cells to produce lupus autoantibodies in vivo. Eur J Immunol. 1995;25:3412–7. 8566031 10.1002/eji.1830251231

[7] Fairhurst AM, Hwang SH, Wang A, Tian XH, Boudreaux C, Zhou XJ, et al Yaa autoimmune phenotypes are conferred by overexpression of TLR7. Eur J Immunol. 2008;38:1971–8. 10.1002/eji.200838138 18521959 PMC2993003

[8] Elkon KB, Wiedeman A. Type I IFN system in the development and manifestations of SLE. Curr Opin Rheumatol. 2012;:499–505. 22832823 10.1097/BOR.0b013e3283562c3e

[9] Kawai T, Akira S. Toll-like receptor and RIG-I-like receptor signaling. Ann N Y Acad Sci. 2008;1143:1–20. 10.1196/annals.1443.020 19076341

[10] Rubtsov AV, Rubtsova K, Kappler JW, Marrack P. TLR7 drives accumulation of ABCs and autoantibody production in autoimmune-prone mice. Immunol Res. 2013;55:210–6. 10.1007/s12026-012-8365-8 22945807 PMC3935605

[11] Meyer-Bahlburg A, Andrews SF, Yu KO, Porcelli SA, Rawlings DJ. Characterization of a late transitional B cell population highly sensitive to BAFF-mediated homeostatic proliferation. J Exp Med. 2008;205:155–68. 10.1084/jem.20071088 18180309 PMC2234381

[12] Hwang IY, Park C, Harrison K, Kehrl JH. TLR4 signaling augments B lymphocyte migration and overcomes the restriction that limits access to germinal center dark zones. J Exp Med. 2009;206:2641–57. 10.1084/jem.20091982 19917774 PMC2806604

[13] Gitlin AD, Shulman Z, Nussenzweig MC. Clonal selection in the germinal centre by regulated proliferation and hypermutation. Nature. 2014;509:637–40. 10.1038/nature13300 24805232 PMC4271732

[14] Jackson SW, Scharping NE, Kolhatkar NS, Khim S, Schwartz MA, Li QZ, et al Opposing impact of B cell-intrinsic TLR7 and TLR9 signals on autoantibody repertoire and systemic inflammation. J Immunol. 2014;192:4525–32. 10.4049/jimmunol.1400098 24711620 PMC4041708

[15] Soni C, Wong EB, Domeier PP, Khan TN, Satoh T, Akira S, et al B Cell-Intrinsic TLR7 Signaling Is Essential for the Development of Spontaneous Germinal Centers. J Immunol. 2014;193:4400–14. 10.4049/jimmunol.1401720 25252960 PMC4201954

[16] Subramanian S, Tus K, Li QZ, Wang A, Tian XH, Zhou J, et al A Tlr7 translocation accelerates systemic autoimmunity in murine lupus. Proc Natl Acad Sci U S A. 2006;103:9970–5. 16777955 10.1073/pnas.0603912103 PMC1502563

[17] Pisitkun P, Deane JA, Difilippantonio MJ, Tarasenko T, Satterthwaite AB, Bolland S. Autoreactive B cell responses to RNA-related antigens due to TLR7 gene duplication. Science. 2006;312:1669–72. 16709748 10.1126/science.1124978

[18] Kahn P, Ramanujam M, Bethunaickan R, Huang W, Tao H, Madaio MP, et al Prevention of murine antiphospholipid syndrome by BAFF blockade. Arthritis Rheum. 2008;58:2824–34. 10.1002/art.23764 18759321 PMC2596604

[19] Deane JA, Pisitkun P, Barrett RS, Feigenbaum L, Town T, Ward JM, et al Control of toll-like receptor 7 expression is essential to restrict autoimmunity and dendritic cell proliferation. Immunity. 2007;27:801–10. 17997333 10.1016/j.immuni.2007.09.009 PMC2706502

[20] Santiago-Raber M, Kijuchi S, Borel P, Uematsu S, Akira S, Kotzin B, et al Evidence for Genes in Addition to *Tlr7* in the *Yaa* Translocation Linked with Acceleration of Systemic Lupus Erythematosus. J Immunol. 2008;181:1556–62 18606711 10.4049/jimmunol.181.2.1556

[21] Moisini I, Huang W, Bethunaickan R, Sahu R, Ricketts PG, Akerman M, et al The Yaa locus and IFN-alpha fine-tune germinal center B cell selection in murine systemic lupus erythematosus. J Immunol. 2012;189:4305–12. 10.4049/jimmunol.1200745 23024275 PMC3478483

[22] Chen C, Nagy Z, Prak EL, Weigert M. Immunoglobulin heavy chain gene replacement: a mechanism of receptor editing. Immunity. 1995;3:747–55. 8777720 10.1016/1074-7613(95)90064-0

[23] Ramanujam M, Kahn P, Huang W, Tao H, Madaio MP, Factor SM, et al Interferon-alpha treatment of female (NZW x BXSB)F(1) mice mimics some but not all features associated with the Yaa mutation. Arthritis Rheum. 2009;60:1096–101. 10.1002/art.24414 19333924 PMC2703814

[24] Wang X, Huang W, Mihara M, Sinha J, Davidson A. Mechanism of action of combined short-term CTLA4Ig and anti-CD40 ligand in murine systemic lupus erythematosus. J Immunol. 2002;168:2046–53. 11823542 10.4049/jimmunol.168.4.2046

[25] Akkerman A, Huang W, Wang X, Ramanujam M, Schiffer L, Madaio M, et al CTLA4Ig prevents initiation but not evolution of anti-phospholipid syndrome in NZW/BXSB mice. Autoimmunity. 2004;37:445–51. 15621570 10.1080/08916930400008524 PMC2701307

[26] Ramanujam M, Wang X, Huang W, Schiffer L, Grimaldi C, Akkerman A, et al Mechanism of action of transmembrane activator and calcium modulator ligand interactor-Ig in murine systemic lupus erythematosus. J Immunol. 2004;173:3524–34. 15322217 10.4049/jimmunol.173.5.3524

[27] Ramanujam M, Wang X, Huang W, Liu Z, Schiffer L, Tao H, et al Similarities and differences between selective and nonselective BAFF blockade in murine SLE. J Clin Invest. 2006;116:724–34. 16485042 10.1172/JCI26385 PMC1366500

[28] Liu Z, Bethunaickan R, Huang W, Lodhi U, Solano I, Madaio MP, et al Interferon-alpha accelerates murine systemic lupus erythematosus in a T cell-dependent manner. Arthritis Rheum. 2011;63:219–29. 10.1002/art.30087 20954185 PMC3014995

[29] Wardemann H, Yurasov S, Schaefer A, Young JW, Meffre E, Nussenzweig MC. Predominant autoantibody production by early human B cell precursors. Science. 2003;301:1374–7. 12920303 10.1126/science.1086907

[30] Schiffer L, Bethunaickan R, Ramanujam M, Huang W, Schiffer M, Tao H, et al Activated renal macrophages are markers of disease onset and disease remission in lupus nephritis. J Immunol. 2008;180:1938–47. 18209092 10.4049/jimmunol.180.3.1938 PMC2587994

[31] Huang W, Moisini I, Bethunaickan R, Sahu R, Akerman M, Eilat D, et al BAFF/APRIL inhibition decreases selection of naive but not antigen-induced autoreactive B cells in murine systemic lupus erythematosus. J Immunol. 2011;187:6571–80. 10.4049/jimmunol.1101784 22102726 PMC3263361

[32] Arango D, Wilson AJ, Shi Q, Corner GA, Aranes MJ, Nicholas C, et al Molecular mechanisms of action and prediction of response to oxaliplatin in colorectal cancer cells. Br J Cancer. 2004;91:1931–46. 15545975 10.1038/sj.bjc.6602215 PMC2409767

[33] Gentleman R, Carey V, Huber W, Irizarry R, Dudoit S. Bioinformatics and Computational Biology Solutions Using R and Bioconductor: Springer Science+Business Media, Inc; 2005 p. 161–79.

[34] Simon RM, Korn EL, McShane LM, Radmacher MD, Wright GW, Zhao Y. Design and Analysis of DNA Microarray Investigations: Springer Science+Business Media, Inc; 2004 p. 121–55.

[35] Lesser ML, M A . An Exploratory Graphical Method for Identifying Associations in r x c Contingency Tables. Journal of Modern Applied Statistical Methods. 2014;13:91–109.

[36] Alabyev B, Rahman ZS, Manser T. Quantitatively reduced participation of anti-nuclear antigen B cells that down-regulate B cell receptor during primary development in the germinal center/memory B cell response to foreign antigen. J Immunol. 2007;178:5623–34. 17442945 10.4049/jimmunol.178.9.5623

[37] Kirchenbaum GA, St Clair JB, Detanico T, Aviszus K, Wysocki LJ. Functionally responsive self-reactive B cells of low affinity express reduced levels of surface IgM. Eur J Immunol. 2014;44:970–82. 10.1002/eji.201344276 24375379 PMC3984621

[38] Radic MZ, Mackle J, Erikson J, Mol C, Anderson WF, Weigert M. Residues that mediate DNA binding of autoimmune antibodies. J Immunol. 1993;150:4966–77. 8496598

[39] Radic MZ, Mascelli MA, Erikson J, Shan H, Weigert M. Ig H and L chain contributions to autoimmune specificities. J Immunol. 1991;146:176–82. 1898596

[40] Radic MZ, Weigert M. Genetic and structural evidence for antigen selection of anti-DNA antibodies. Annu Rev Immunol. 1994;12:487–520. 8011289 10.1146/annurev.iy.12.040194.002415

[41] Ibrahim SM, Weigert M, Basu C, Erikson J, Radic MZ. Light chain contribution to specificity in anti-DNA antibodies. J Immunol. 1995;155:3223–33. 7673735

[42] Radic MZ, Erikson J, Litwin S, Weigert M. B lymphocytes may escape tolerance by revising their antigen receptors. J Exp Med. 1993;177:1165–73. 8459210 10.1084/jem.177.4.1165 PMC2190988

[43] Mandik-Nayak L, Bui A, Noorchashm H, Eaton A, Erikson J. Regulation of anti-double-stranded DNA B cells in nonautoimmune mice: localization to the T-B interface of the splenic follicle. J Exp Med. 1997;186:1257–67. 9334365 10.1084/jem.186.8.1257 PMC2199093

[44] Paul E, Lutz J, Erikson J, Carroll MC. Germinal center checkpoints in B cell tolerance in 3H9 transgenic mice. Int Immunol. 2004;16:377–84. 14734623 10.1093/intimm/dxh035

[45] Li Y, Li H, Ni D, Weigert M. Anti-DNA B cells in MRL/lpr mice show altered differentiation and editing pattern. J Exp Med. 2002;196:1543–52. 12486097 10.1084/jem.20021560 PMC2196070

[46] Koh YT, Scatizzi JC, Gahan JD, Lawson BR, Baccala R, Pollard KM, et al Role of nucleic acid-sensing TLRs in diverse autoantibody specificities and anti-nuclear antibody-producing B cells. J Immunol. 2013;190:4982–90. 10.4049/jimmunol.1202986 23589617 PMC3729324

[47] Hua Z, Gross AJ, Lamagna C, Ramos-Hernandez N, Scapini P, Ji M, et al Requirement for MyD88 signaling in B cells and dendritic cells for germinal center anti-nuclear antibody production in Lyn-deficient mice. J Immunol. 2014;192:875–85. 10.4049/jimmunol.1300683 24379120 PMC4101002

[48] Teichmann LL, Schenten D, Medzhitov R, Kashgarian M, Shlomchik MJ. Signals via the adaptor MyD88 in B cells and DCs make distinct and synergistic contributions to immune activation and tissue damage in lupus. Immunity. 2013;38:528–40. 10.1016/j.immuni.2012.11.017 23499488 PMC3638041

[49] Sabouri Z, Schofield P, Horikawa K, Spierings E, Kipling D, Randall KL, et al Redemption of autoantibodies on anergic B cells by variable-region glycosylation and mutation away from self-reactivity. Proc Natl Acad Sci U S A. 2014;111:E2567–75. 10.1073/pnas.1406974111 24821781 PMC4078846

[50] Clingan JM, Matloubian M. B Cell-intrinsic TLR7 signaling is required for optimal B cell responses during chronic viral infection. J Immunol. 2013;191:810–8. 10.4049/jimmunol.1300244 23761632 PMC3702634

