# Supplementary figures and images for: TLR7 Influences Germinal Center Selection in Murine SLE
Source: PLoS One. 2015 Mar 20;10(3):e0119925. doi: 10.1371/journal.pone.0119925 (PMC4368537; doi:10.1371/journal.pone.0119925)

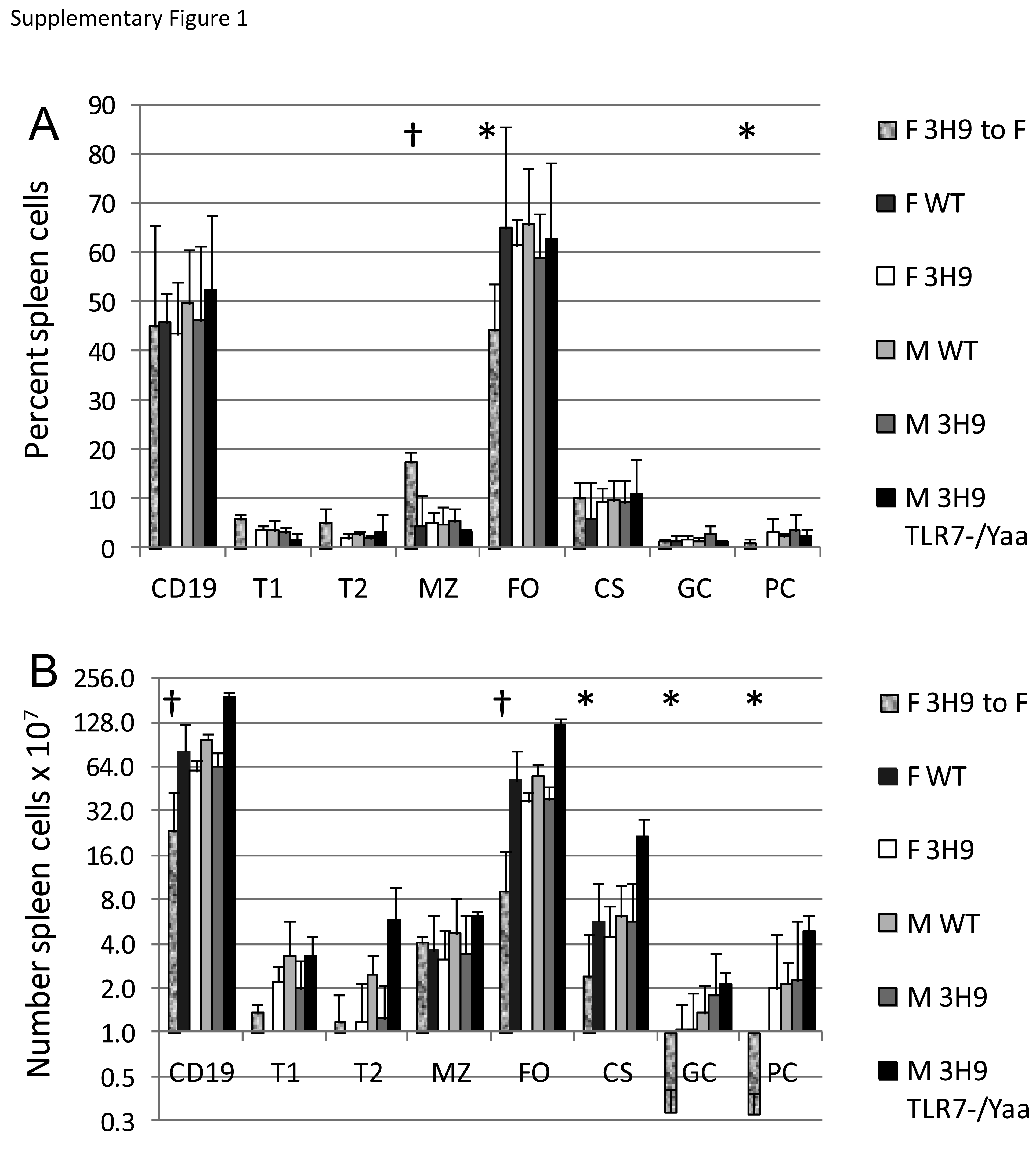

Supplement: S1 Fig — The 3H9 F to F chimeras have a higher percentage of marginal zone (MZ) B cells and a lower total number of follicular (FO), class switched (CS), germinal center (GC) and plasma cells (PC) than all the other chimeras. Comparisons are with male WT chimeras. n = 5–12 per group. * p < 0.05, † p < 0.01. (TIF) [file pone.0119925.s001.tif]
