# Supplementary material for: TLR7 Influences Germinal Center Selection in Murine SLE
Source: PLoS One. 2015 Mar 20;10(3):e0119925. doi: 10.1371/journal.pone.0119925 (PMC4368537; doi:10.1371/journal.pone.0119925)
Supplement: S1 Table — Generation of mouse strains used for analysis of the TLR7-/Yaa phenotype and as donors for the bone marrow chimeras. (DOCX) [file pone.0119925.s002.docx]

**Supplementary Table 1**: Experimental mouse strains

| **Parental strain**  **Female** | **Parental strain**  **Male** | | | | | **F1** | | | **Use** |  |
| --- | --- | --- | --- | --- | --- | --- | --- | --- | --- | --- |
|  |  |  |  |  |  |  |  |  |  |  |
| NZW TLR7^+/-^ | BXSB | | | | | NZW/BXSB ^TLR7-/Yaa^ or  NZW/BXSB wt | | | Figures 1, 2 |  |
|  |  |  | |  |  | | |  | | |
| 3H9^+/+^ NZW |  | BXSB | |  | 3H9 NZW/BXSB M or | | | Donors for | | |
|  |  |  |  | | 3H9 NZW/BXSB F | | | chimeras | | |
|  |  |  | | |  | | |  | | |
| 3H9^+/+^ NZW TLR7^-/-^ |  | BXSB | | | 3H9 NZW/BXSB ^TLR7-/Yaa^ M | | | Donors for chimeras | | |
|  |  |  |  | |  | |  |  | | |
