# Supplementary material for: TLR7 Influences Germinal Center Selection in Murine SLE
Source: PLoS One. 2015 Mar 20;10(3):e0119925. doi: 10.1371/journal.pone.0119925 (PMC4368537; doi:10.1371/journal.pone.0119925)
Supplement: S2 Table — The Table shows the number of Vκ genes represented in each comparison, the number of genes that contribute the top 50% of the statistical difference in the λ 2 analysis and the percent contribution of the most differentially expressed gene. Individual genes that contribute >5% of the statistical difference in the λ 2 analysis are shown on the right for each comparison. See methods section for a description of the statistical analysis. (DOCX) [file pone.0119925.s003.docx]

| **B cell Subset Comparison** | | | **No. Vκ genes** | **No. genes in top 50%†** | **Top gene**‡  **(% λ^2^)** | **Individual genes contributing > 5% to the λ^2^ analysis** | | | | | | | | |
| --- | --- | --- | --- | --- | --- | --- | --- | --- | --- | --- | --- | --- | --- | --- |
| F 3H9 | M 3H9 | FO | 58 | 9 | 9.8 | 9-123 | 6-25 | 4-55 | 4-57-1 | 3-10 |  |  |  |  |
| TLR7^-/Yaa^ | M 3H9 | FO | 54 | 9 | 11.3 | 8-24 | 9-123 | 12-98 | 3-7 | 4-74 |  |  |  |  |
| F 3H9 | TLR7^-/Yaa^ | FO | 52 | 9 | 15.7 | 3-12 | 13-84/85 | 1-99 |  |  |  |  |  |  |
| F 3H9 to F | TLR7^-/Yaa^ | FO | 52 | 9 | 9.5 | 3-12 | 13-84/85 | 10-94 | 3-5 | 3-3 | 8-28 |  |  |  |
| F 3H9 to F | F 3H9 | FO | 55 | 10 | 9.9 | 8-24 | 3-3 | 3-5 | 6-25 | 13-84/85 | 3-12 |  |  |  |
| F 3H9 to F | M 3H9 | FO | 45 | 7 | 12.8 | 8-24 | 10-94 | 1-117 | 3-3 | 13-84/85 | 3-5 |  |  |  |
|  |  |  |  |  |  |  |  |  |  |  |  |  |  |  |
| F 3H9 | M 3H9 | GC | 41 | 8 | 9.7 | 8-24 | 3-12 | 3-10 | 10-94 |  |  |  |  |  |
| TLR7^-/Yaa^ | M 3H9 | GC | 41 | 12 | 8.1 | 10-96 | 4-74 |  |  |  |  |  |  |  |
| F 3H9 | TLR7^-/Yaa^ | GC | 41 | 10 | 7.2 | 13-84/85 | 4-74 | 1-117 | 3-2 | 6-15 |  |  |  |  |
| F 3H9 to F | TLR7^-/Yaa^ | GC | 34 | 7 | 14.2 | 5-43/45 | 3-12 | 1-110 | 6-15 | 3-5 |  |  |  |  |
| F 3H9 to F | F 3H9 | GC | 41 | 5 | 15.8 | 3-12 | 13-84 | 5-43/45 | 1-117 | 3-5 | 4-61 |  |  |  |
| F 3H9 to F | M 3H9 | GC | 41 | 4 | 27.2 | 3-12 | 5-43/45 | 3-5 | 10-96 | 9-120 |  |  |  |  |
|  |  |  |  |  |  |  |  |  |  |  |  |  |  |  |
| F 3H9 | M 3H9 | PC | 35 | 6 | 13.5 | 3-12 | 3-1 | 1-117 | 3-10 | 5-43/45 | 6-23 |  |  |  |
| M 3H9 | TLR7^-/Yaa^ | PC | 35 | 9 | 12.9 | 5-43/45 | 3-1 | 10-96 | 3-5 |  |  |  |  |  |
| F 3H9 | TLR7^-/Yaa^ | PC | 35 | 6 | 17.5 | 10-96 | 3-5 | 3-12 | 1-117 | 4-53 |  |  |  |  |
| F 3H9 to F | TLR7^-/Yaa^ | PC | 41 | 5 | 15.2 | 16-104 | 5-43/45 | 9-124 | 1-110 | 6-15 |  |  |  |  |
| F 3H9 to F | F 3H9 | PC | 20 | 5 | 22.6 | 5-43/45 | 9-120 | 10-96 | 6-15 | 1-110 | 16-104 | 5-48 | 3-5 | 4-57-1 |
| F 3H9 to F | M 3H9 | PC | 33 | 4 | 34.5 | 5-43/45 | 9-120 | 3-1 |  |  |  |  |  |  |

**Supplementary Table 2**: Pairwise comparisons of Vκ repertoires of FO, GC and PC subsets*

* See reference 35

† Number of genes contributing the top 50% to the overall λ^2^

‡ Percent contribution of the top gene to the overall λ^2^
