# Supplementary material for: TLR7 Influences Germinal Center Selection in Murine SLE
Source: PLoS One. 2015 Mar 20;10(3):e0119925. doi: 10.1371/journal.pone.0119925 (PMC4368537; doi:10.1371/journal.pone.0119925)
Supplement: S3 Table — Vκ usage of each 3H9-expressing single cells sorted from each of the follicular, germinal center and plasma cell subsets of bone marrow chimeric mice. The number of cells expressing each Vκ is shown for each set of chimeras. See Fig. 6 for graphical representation of selected genes. (DOCX) [file pone.0119925.s004.docx]

**Supplementary Table 3**: 3H9 associated Vκ usage of B cell subsets from chimeric mice

| **Follicular B cells** | | | | | |
| --- | --- | --- | --- | --- | --- |
| **Vκ gene** | **V allele** | **M 3H9** | **F 3H9** | **TLR7^-^/Yaa** | **F 3H9 to F** |
| 1 | 110*01 | 5 | 3 | 3 | 0 |
| 1 | 117*01 | 0 | 4 | 2 | 2 |
| 1 | 135*01 | 2 | 3 | 2 | 0 |
| 1 | 88*01 | 1 | 2 | 0 | 1 |
| 1 | 99*01 | 3 | 3 | 1 | 2 |
| 2 | 109*01 | 0 | 0 | 1 | 1 |
| 2 | 137*01 | 0 | 0 | 1 | 1 |
| 2 | 112*01 | 1 | 0 | 0 | 0 |
| 3 | 1*01 | 5 | 9 | 1 | 2 |
| 3 | 10*01 | 19 | 10 | 10 | 3 |
| 3 | 12*01 | 9 | 9 | 3 | 8 |
| 3 | 2*01 | 8 | 11 | 3 | 3 |
| 3 | 3*01 | 0 | 0 | 0 | 2 |
| 3 | 4*01 | 21 | 12 | 9 | 6 |
| 3 | 5*01 | 8 | 9 | 5 | 9 |
| 3 | 7*01 | 0 | 4 | 3 | 0 |
| 4 | 53*01 | 0 | 1 | 0 | 0 |
| 4 | 55*01 | 10 | 3 | 7 | 3 |
| 4 | 57*01 | 1 | 2 | 2 | 0 |
| 4 | 57-1*01 | 8 | 2 | 5 | 2 |
| 4 | 58*01 | 1 | 0 | 0 | 0 |
| 4 | 59*01 | 0 | 1 | 2 | 0 |
| 4 | 63*01 | 0 | 4 | 1 | 0 |
| 4 | 69*01 | 0 | 1 | 0 | 0 |
| 4 | 70*01 | 0 | 1 | 0 | 1 |
| 4 | 72*01 | 0 | 0 | 1 | 0 |
| 4 | 74*01 | 0 | 0 | 3 | 0 |
| 4 | 79*01 | 0 | 1 | 0 | 0 |
| 4 | 80*01 | 0 | 1 | 0 | 0 |
| 4 | 86*01 F | 0 | 1 | 0 | 0 |
| 4 | 91*01 | 1 | 0 | 1 | 1 |
| 5 | 39*01 | 4 | 4 | 1 | 1 |
| 5 | 43/45*01 | 1 | 3 | 2 | 2 |
| 5 | 48*01 | 2 | 0 | 2 | 0 |
| 6 | 13*01 | 0 | 0 | 1 | 0 |
| 6 | 14*01 | 1 | 0 | 0 | 0 |
| 6 | 15*01 | 1 | 1 | 1 | 2 |
| 6 | 17*01 | 3 | 2 | 2 | 1 |
| 6 | 20*01 | 0 | 1 | 1 | 0 |
| 6 | 23*01 | 4 | 6 | 2 | 2 |
| 6 | 25*01 | 2 | 11 | 2 | 1 |
| 6 | 32*01 | 1 | 0 | 0 | 0 |
| 8 | 18*01 | 0 | 1 | 2 | 1 |
| 8 | 19*01 | 4 | 3 | 1 | 1 |
| 8 | 21*01 | 1 | 2 | 0 | 1 |
| 8 | 24*01 | 0 | 1 | 6 | 4 |
| 8 | 27*01 | 1 | 3 | 3 | 0 |
| 8 | 28*01 | 6 | 6 | 5 | 1 |
| 8 | 30*01 | 4 | 5 | 7 | 2 |
| 9 | 120*01 | 2 | 0 | 0 | 1 |
| 9 | 123*01 | 7 | 0 | 0 | 1 |
| 10 | 94*01 | 4 | 10 | 4 | 8 |
| 10 | 96*01 | 0 | 0 | 1 | 0 |
| 11 | 125*01 | 0 | 1 | 0 | 0 |
| 12 | 38*01 | 2 | 3 | 1 | 2 |
| 12 | 41*01 | 0 | 3 | 1 | 0 |
| 12 | 44*01 | 4 | 10 | 6 | 5 |
| 12 | 46*01 | 11 | 19 | 5 | 4 |
| 12 | 98*01 | 0 | 2 | 4 | 0 |
| 13 | 84/85*01 | 27 | 25 | 23 | 5 |
| 14 | 100*01 | 1 | 2 | 2 | 2 |
| 14 | 130*01 | 0 | 2 | 0 | 0 |
| 16 | 104*01 | 2 | 6 | 2 | 3 |
| 19 | 93*01 | 2 | 0 | 0 | 0 |
|  | Total | 200 | 229 | 153 | 97 |
| **Germinal center B cells** | | | | | |
| **Vκ gene** | **V allele** | **M 3H9** | **F 3H9** | **TLR7^-^/Yaa** | **F 3H9 to F** |
| 1 | 110*01 | 3 | 5 | 3 | 0 |
| 1 | 117*01 | 1 | 10 | 1 | 0 |
| 1 | 133*01 | 1 | 2 | 0 | 0 |
| 1 | 135*01 | 2 | 3 | 0 | 0 |
| 1 | 88*01 | 1 | 0 | 1 | 0 |
| 1 | 99*01 | 0 | 1 | 1 | 0 |
| 2 | 137*01 | 2 | 2 | 0 | 0 |
| 3 | 1*01 | 4 | 0 | 0 | 0 |
| 3 | 12*01 | 1 | 3 | 2 | 6 |
| 3 | 2*01 | 2 | 1 | 3 | 1 |
| 3 | 3*01 | 2 | 0 | 1 | 0 |
| 3 | 4*01 | 8 | 0 | 1 | 0 |
| 3 | 5*01 | 2 | 2 | 1 | 3 |
| 3 | 7*01 | 0 | 4 | 0 | 0 |
| 4 | 50*01 | 0 | 0 | 0 | 0 |
| 4 | 55*01 | 0 | 0 | 0 | 0 |
| 4 | 52*01 | 1 | 0 | 0 | 0 |
| 4 | 57-1*01 | 9 | 3 | 1 | 2 |
| 4 | 58*01 | 0 | 0 | 1 | 0 |
| 4 | 59*01 | 1 | 1 | 0 | 1 |
| 4 | 60*01 | 0 | 1 | 0 | 0 |
| 4 | 61*01 | 0 | 0 | 1 | 1 |
| 4 | 69*01 | 0 | 1 | 0 | 0 |
| 4 | 78*01 | 0 | 1 | 0 | 0 |
| 4 | 91*01 | 0 | 1 | 0 | 0 |
| 4 | 70*01 | 0 | 0 | 0 | 0 |
| 4 | 74*01 | 0 | 0 | 2 | 0 |
| 4 | 79*01 | 0 | 0 | 1 | 0 |
| 4 | 61*01 | 2 | 0 | 0 | 0 |
| 4 | 63*01 | 2 | 0 | 0 | 0 |
| 4 | 73*01 | 3 | 0 | 0 | 0 |
| 4 | 74*01 | 1 | 0 | 0 | 0 |
| 4 | 80*01 | 1 | 0 | 1 | 0 |
| 5 | 39*01 | 1 | 5 | 1 | 1 |
| 5 | 43/45*01 | 52 | 45 | 21 | 27 |
| 5 | 48*01 | 35 | 22 | 13 | 5 |
| 6 | 15*01 | 12 | 1 | 3 | 0 |
| 6 | 17*01 | 1 | 1 | 0 | 0 |
| 6 | 20*01 | 0 | 0 | 0 | 0 |
| 6 | 23*01 | 4 | 3 | 1 | 0 |
| 6 | 25*01 | 1 | 1 | 0 | 1 |
| 6 | 32*01 | 0 | 1 | 0 | 0 |
| 8 | 21*01 | 11 | 1 | 0 | 0 |
| 8 | 24*01 | 0 | 1 | 2 | 0 |
| 8 | 27*01 | 0 | 0 | 0 | 0 |
| 8 | 28*01 | 0 | 1 | 1 | 0 |
| 8 | 30*01 | 2 | 1 | 0 | 0 |
| 9 | 120*01 | 14 | 2 | 2 | 0 |
| 9 | 123*01 | 1 | 0 | 0 | 0 |
| 9 | 124*01 | 5 | 0 | 0 | 0 |
| 10 | 94*01 | 2 | 1 | 2 | 0 |
| 10 | 96*01 | 0 | 2 | 2 | 1 |
| 12 | 38*01 | 1 | 1 | 0 | 0 |
| 12 | 44*01 | 0 | 3 | 1 | 0 |
| 12 | 46*01 | 1 | 2 | 2 | 1 |
| 12 | 98*01 | 0 | 0 | 1 | 0 |
| 13 | 84/85*01 | 3 | 1 | 2 | 2 |
| 14 | 100*01 | 1 | 1 | 1 | 0 |
| 14 | 111*01 | 1 | 1 | 0 | 0 |
| 16 | 104*01 | 3 | 2 | 1 | 0 |
|  | Total | 200 | 140 | 77 | 52 |
| **Plasma cells** | | | | | |
| **Vκ gene** | **V allele** | **M 3H9** | **F 3H9** | **TLR7^-^/Yaa** | **F 3H9 to F** |
| 1 | 35*01 | 0 | 0 | 1 | 0 |
| 1 | 110*1 | 2 | 0 | 1 | 2 |
| 1 | 117*01 | 0 | 3 | 2 | 2 |
| 1 | 88*01 | 1 | 0 | 0 | 0 |
| 1 | 132*01 | 0 | 0 | 1 | 0 |
| 1 | 135*01 | 0 | 0 | 3 | 0 |
| 3 | 1*01 | 6 | 0 | 1 | 0 |
| 3 | 10*01 | 4 | 0 | 3 | 0 |
| 3 | 12*01 | 0 | 4 | 3 | 1 |
| 3 | 2*01 | 2 | 0 | 3 | 0 |
| 3 | 4*01 | 2 | 1 | 7 | 0 |
| 3 | 5*01 | 2 | 2 | 0 | 0 |
| 3 | 7*01 | 1 | 0 | 4 | 0 |
| 3 | 9*01 | 0 | 1 | 1 | 0 |
| 4 | 50*01 | 0 | 0 | 4 | 0 |
| 4 | 53*01 | 0 | 1 | 0 | 0 |
| 4 | 55*01 | 0 | 0 | 1 | 0 |
| 4 | 57-1*01 | 1 | 2 | 3 | 0 |
| 4 | 58*01 | 1 | 0 | 0 | 0 |
| 4 | 61*01 | 0 | 0 | 1 | 1 |
| 4 | 63*01 | 1 | 0 | 0 | 0 |
| 4 | 68*01 | 0 | 1 | 0 | 0 |
| 4 | 69*01 | 0 | 0 | 2 | 0 |
| 4 | 72*01 | 0 | 1 | 0 | 0 |
| 4 | 74*01 | 0 | 1 | 0 | 0 |
| 4 | 79*01 | 0 | 0 | 2 | 0 |
| 4 | 80*01 | 0 | 0 | 3 | 0 |
| 4 | 91*01 | 1 | 0 | 1 | 0 |
| 5 | 39*01 | 1 | 1 | 2 | 2 |
| 5 | 43/45*01 | 16 | 19 | 85 | 39 |
| 5 | 48*01 | 11 | 8 | 15 | 3 |
| 6 | 15*01 | 2 | 3 | 8 | 0 |
| 6 | 17*01 | 1 | 0 | 0 | 0 |
| 6 | 20*01 | 0 | 0 | 1 | 0 |
| 6 | 23*01 | 3 | 0 | 2 | 0 |
| 6 | 25*01 | 2 | 0 | 0 | 0 |
| 6 | 32*01 | 2 | 0 | 1 | 0 |
| 8 | 18*01 | 1 | 0 | 0 | 0 |
| 8 | 21*01 | 0 | 0 | 2 | 0 |
| 8 | 28*01 | 0 | 1 | 1 | 0 |
| 8 | 30*01 | 1 | 0 | 2 | 0 |
| 9 | 120*01 | 7 | 4 | 1 | 0 |
| 9 | 124*01 | 0 | 0 | 0 | 1 |
| 10 | 94*01 | 1 | 1 | 1 | 1 |
| 10 | 96*01 | 2 | 3 | 0 | 0 |
| 12 | 41*01 | 0 | 0 | 1 | 0 |
| 12 | 44*01 | 1 | 0 | 2 | 0 |
| 12 | 46*01 | 1 | 0 | 4 | 0 |
| 13 | 84/85*01 | 2 | 1 | 6 | 1 |
| 14 | 100*01 | 0 | 0 | 1 | 0 |
| 14 | 111*01 | 0 | 0 | 1 | 0 |
| 14 | 130*01 | 1 | 0 | 0 | 0 |
| 15 | 103*01 | 1 | 0 | 0 | 0 |
| 16 | 104*01 | 1 | 0 | 0 | 2 |
|  | Total | 77 | 58 | 183 | 55 |
